# Author Correction: Ketamine disinhibits dendrites and enhances calcium signals in prefrontal dendritic spines

**DOI:** 10.1038/s41467-020-20634-x

**Published:** 2021-01-08

**Authors:** Farhan Ali, Danielle M. Gerhard, Katherine Sweasy, Santosh Pothula, Christopher Pittenger, Ronald S. Duman, Alex C. Kwan

**Affiliations:** 1 Department of Psychiatry, Yale University School of Medicine, New Haven, CT 06511 USA; 2 Child Study Center, Yale University School of Medicine, New Haven, CT 06511 USA; 3 Department of Neuroscience, Yale University School of Medicine, New Haven, CT 06511 USA

**Keywords:** Depression, Dendritic excitability, Inhibition, Inhibition-excitation balance, Spine plasticity

Correction to: *Nature Communications* 10.1038/s41467-019-13809-8, published online 7 January 2020.

The original version of this Article contained an error in Fig. 1g, in which there was an inadvertent duplication of one of the example traces from the set of four cells in the “pre” and “post” conditions. The duplication occurred between the bottom trace under “pre” and the top trace under “post”. The figure has been corrected with different example traces from a set of four cells for each part of the figure. New examples were selected because the authors do not have a record of which cell and at which time points the original example traces were selected from. This error has now been corrected in both the PDF and HTML versions of the Article. The raw data underlying this figure is available as a Source Data file and all data underlying the paper is available at https://github.com/Kwan-Lab/ali2020.

## Source data

Source data

