# Supplementary material for: Ketamine disinhibits dendrites and enhances calcium signals in prefrontal dendritic spines
Source: Nat Commun. 2020 Jan 7;11:72. doi: 10.1038/s41467-019-13809-8 (PMC6946708; doi:10.1038/s41467-019-13809-8)
Supplement: Supplementary file 1 — Supplementary Information [file 41467_2019_13809_MOESM1_ESM.docx]

Supplementary Information

Ketamine disinhibits dendrites and enhances calcium signals

in prefrontal dendritic spines

Ali et al.

**
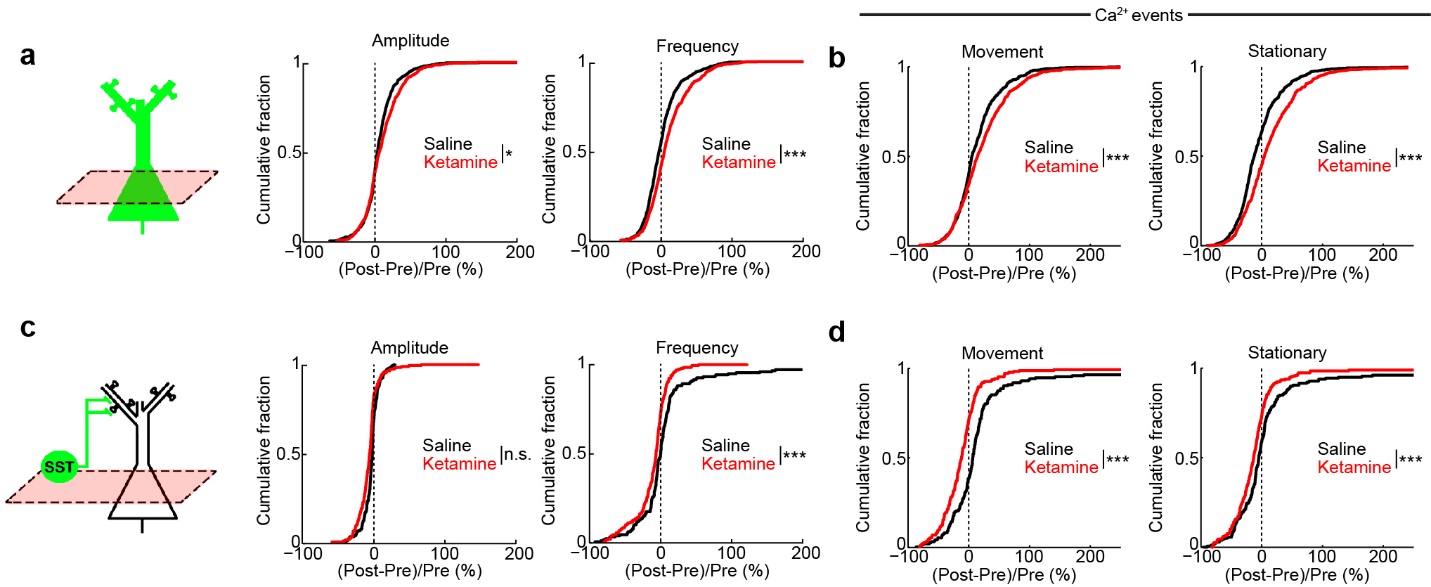
**

**Supplementary Fig. 1 Additional analysis of effects of subanesthetic ketamine on somatic activity of pyramidal neurons and SST interneurons**

(a) Left, Schematic of imaging of pyramidal neurons in Cg1/M2. The normalized difference in amplitude (middle) (ketamine: 11 ± 1%, saline: 7 ± 1%, mean ± s.e.m.; *P* = 0.03, two-sample t-test), and frequency (right) of binned calcium events (ketamine: 10 ± 1%; saline: 1 ± 1%; *P* = 3 x 10^-8^, two-sample t-test) for all data regardless of movement.

(b) Left, the normalized difference in the rate of spontaneous calcium events of pyramidal neurons during movement (ketamine: 25 ± 2%, saline: 15 ± 2%, mean ± s.e.m.; *P* = 9 x 10^-4^, two-sample t-test). Movement was detected by using a threshold calculated by fitting a two-mean Gaussian to the motion trace and taking two standard deviations above the lower mean associated with stationary periods. Any periods with motion estimate above the threshold were considered movement. Right, same as left for stationary periods (ketamine: 23 ± 2%, saline: 5 ± 2%, mean ± s.e.m.; *P* = 8 x 10^-10^, two-sample t-test). For ketamine, *n* = 613 cells from 5 animals. For saline, *n* = 681 cells from 5 animals.

(c) Same as (a) for SST interneurons. Amplitude (ketamine: -4 ± 1%, saline: -1 ± 1%, mean ± s.e.m.; *P* = 0.06, two-sample t-test), and frequency of binned calcium events (ketamine: -10 ± 2%; saline: 12 ± 6%; *P* = 1 x 10^-6^, two-sample t-test).

(d) Same as (b) for SST interneurons (movement: ketamine, -12 ± 3%, saline: 19 ± 6%; *P* = 9 x 10^-4^, two-sample t-test; stationary, ketamine, -13 ± 3%, saline: 9 ± 6%; *P* = 9 x 10^-4^, two-sample t-test). For ketamine, *n* = 198 cells from 5 animals. For saline, *n* = 179 cells from 5 animals.

* *P* < 0.05; ** *P* < 0.01; *** *P* < 0.001; n.s., not significant

**
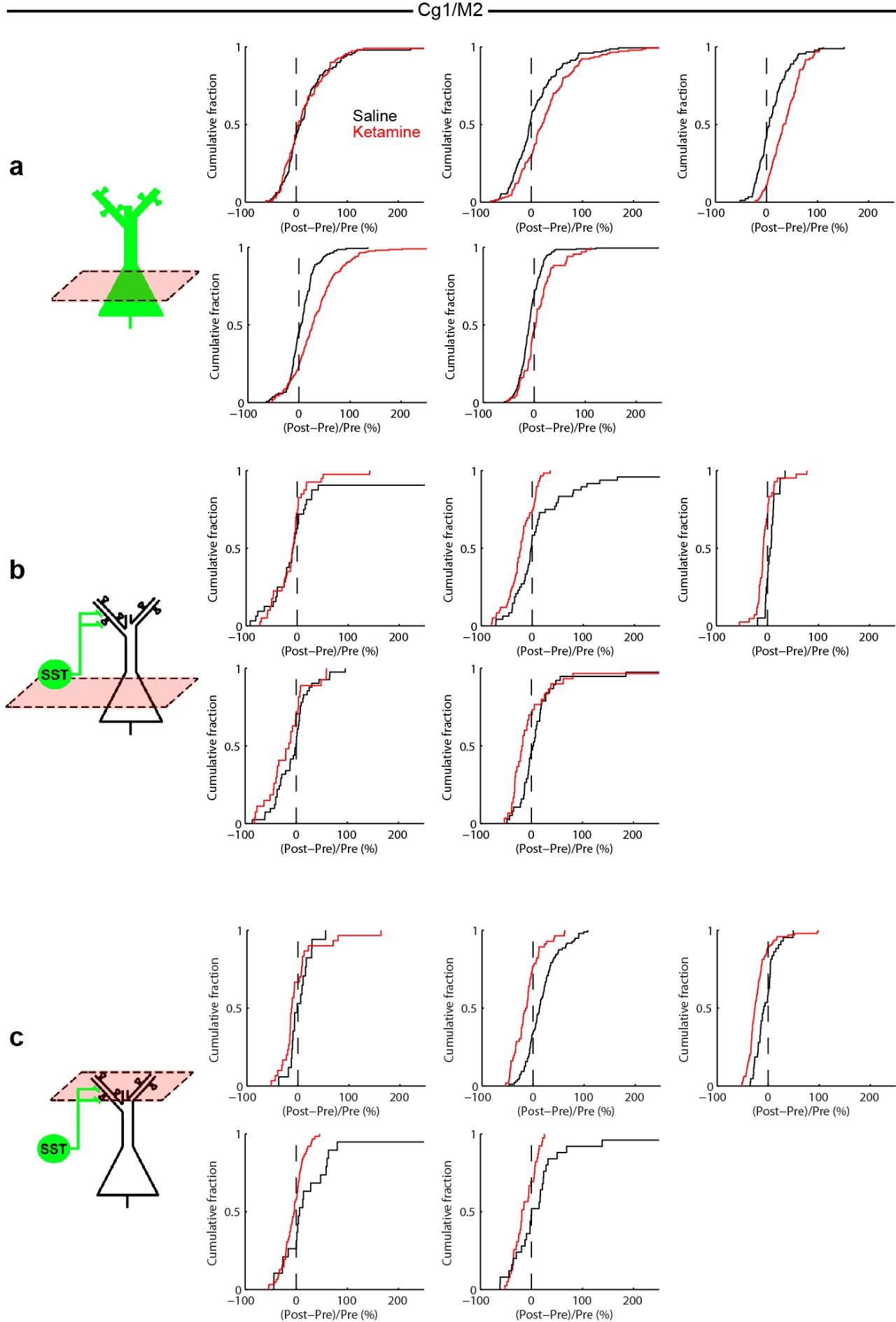

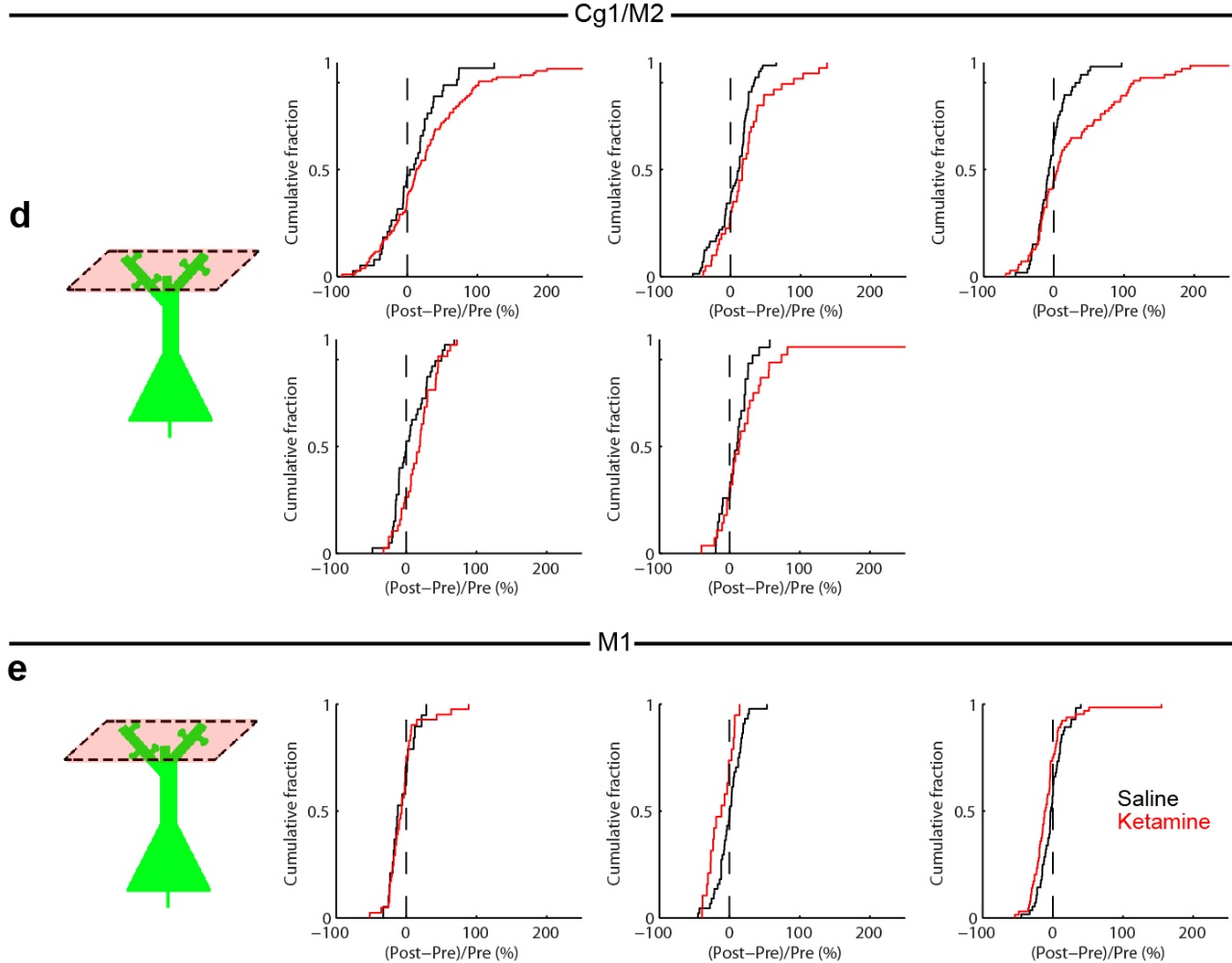
Supplementary Fig. 2 Cumulative fraction plots for individual mice**

(a) Schematic of imaging of pyramidal neurons in Cg1/M2. Each plot is the normalized difference (saline, ketamine) in the rate of spontaneous calcium events for an individual mouse.

(b) Same as (a) for SST interneuron soma in Cg1/M2.

(c) Same as (a) for SST axons in Cg1/M2.

(d) Same as (a) for dendritic spines in Cg1/M2.

(e) Same as (a) for dendritic spines in M1.


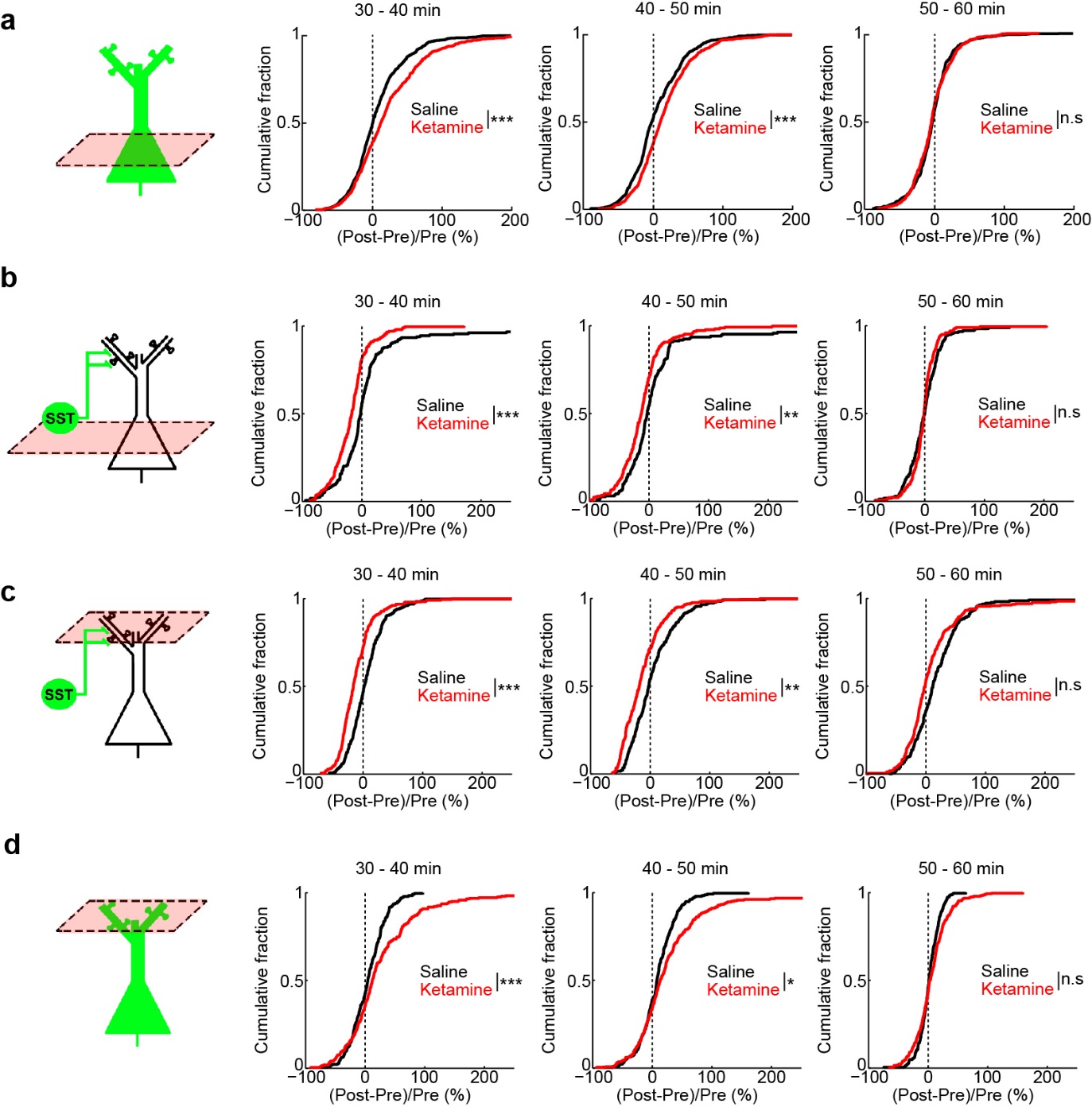


**Supplementary Fig. 3 Time course of effects of ketamine on mPFC activity.**

(a) Left, schematic of imaging of pyramidal neuron in Cg1/M2. Right, the normalized difference in the rate of spontaneous calcium events of pyramidal neurons at 30 – 40 min, 40 – 50 min and 50 – 60 min post-injection respectively.

(b) Same as (a) for SST interneuron.

(c) Same as (a) for SST axons.

(d) Same as (a) for dendritic spines.

* *P* < 0.05; ** *P* < 0.01; *** *P* < 0.001; n.s., not significant, two-sample t-test


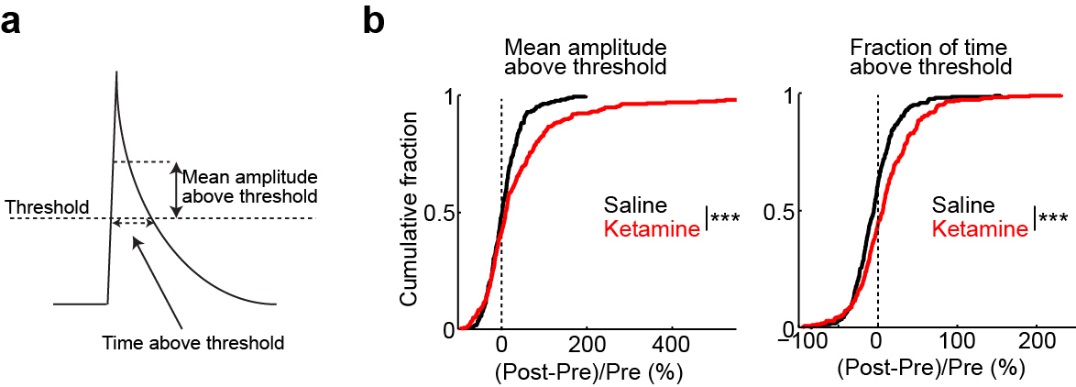


**Supplementary Fig. 4** **Analyzing the calcium dynamics in dendritic spines with an alternative method.**

(a) Schematic illustrating an alternative method to detect calcium events from spine fluorescence transients. For each spine, the threshold was defined as 3 times the median absolute deviation of ΔF/F values across all image frames. We identified image frames in which ΔF/F was above the threshold. From this subset of image frames, we determined the mean amplitude above threshold (Δ*F/F*(*t*) minus the threshold), and the time above the threshold (the number of image frames divided by the frame rate).

(b) Summary of calcium dynamics for apical dendritic spines in Cg1/M2. Left, normalized difference in the mean amplitude above threshold. Normalized difference was calculated as post- minus pre-injection values normalized by the pre-injection value (ketamine: 58 ± 13%, mean ± s.e.m.; saline: 6 ± 3%; *P* = 5 x 10^-4^, two-sample t-test). Right, normalized difference in the fraction of time of an imaging session spent above threshold (ketamine: 10 ± 2%; saline: -3 ± 2%; *P* = 5 x 10^-5^, two-sample t-test). For saline, *n* = 231 dendritic spines from 5 animals. For ketamine, *n* = 280 dendritic spines from 5 animals.

* *P* < 0.05; ** *P* < 0.01; *** *P* < 0.001; n.s., not significant.


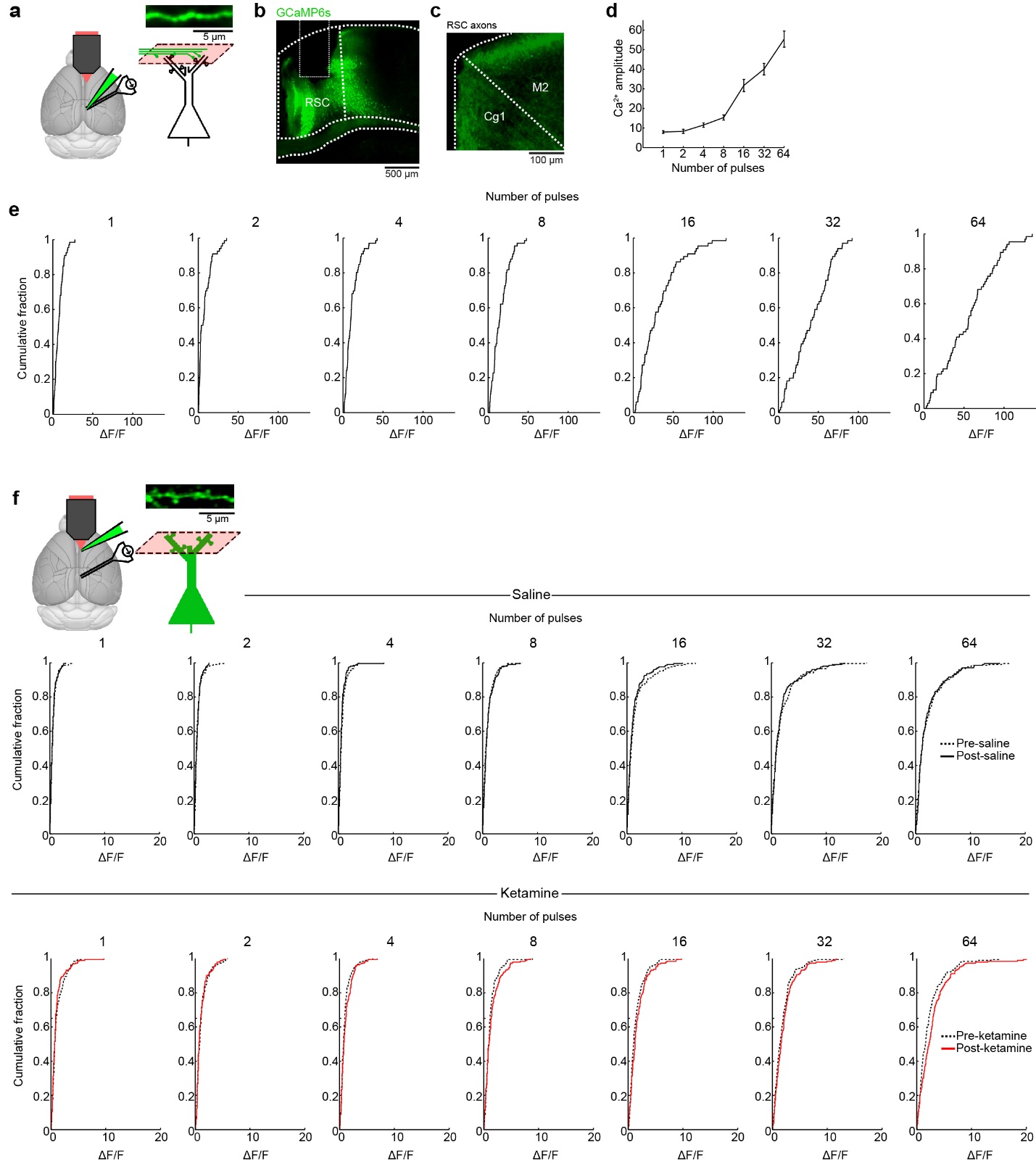


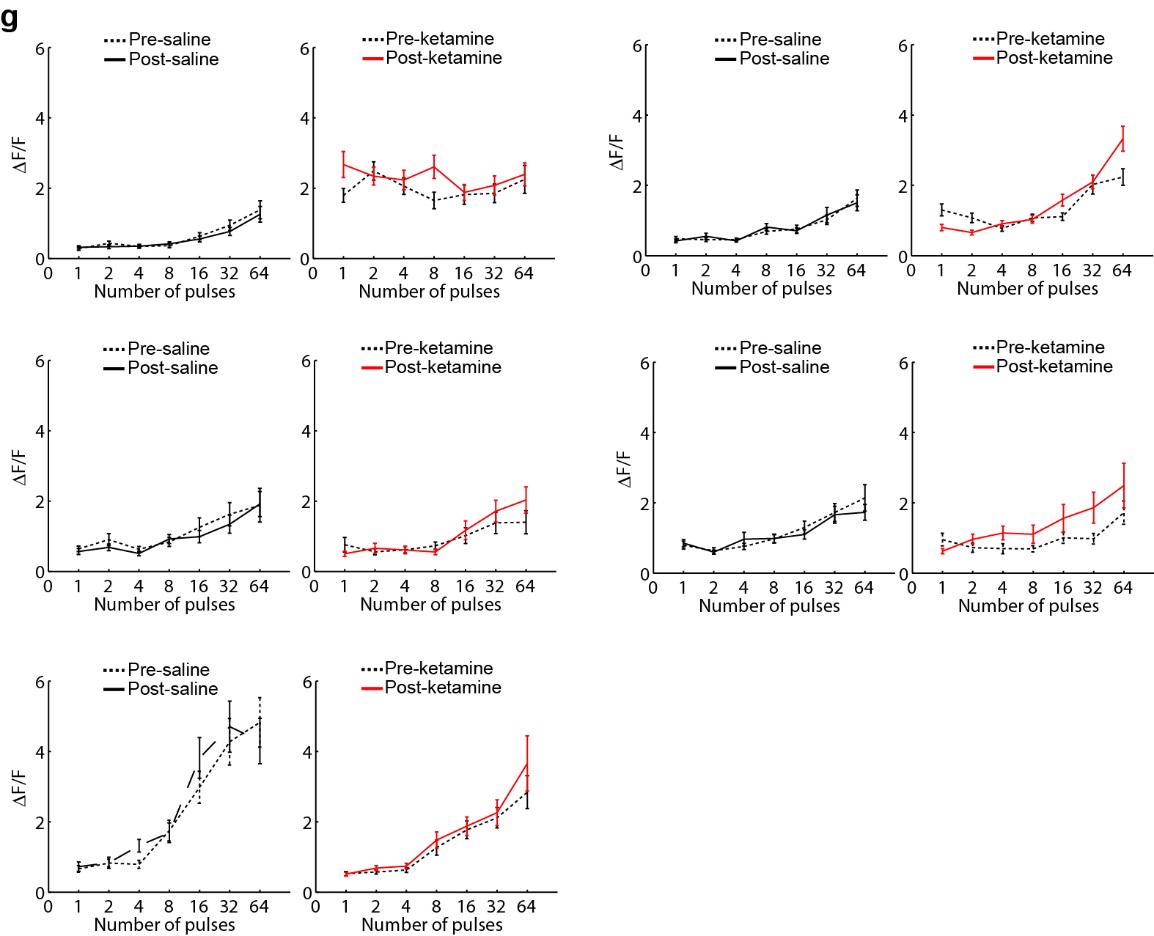


**Supplementary Fig. 5** **Imaging stimulation-induced calcium responses in RSC axons and spines in Cg1/M2.**

(a) Schematic of experimental setup and imaging location. A simulation electrode was placed in the retrosplenial cortex (RSC). A virus was injected to mediate GCaMP6s expression in RSC. Axonal calcium responses were imaged in Cg1/M2.

(b) Coronal histological section, showing the extent of AAV-mediated expression of GCaMP6s. The white rectangle indicates lesion made by insertion of stimulation electrode bundle. RSC, retrosplenial cortex.

(c) Coronal histological section, showing GCaMP6s-expressing axons in the medial prefrontal cortex. Cg1, cingulate cortex. M2, secondary motor cortex.

(d) Trial-averaged calcium responses for RSC axonal boutons, as a function of the number of stimulation pulses applied. Line, mean ± s.e.m. *n* = 66 axonal boutons from 2 animals.

(e) Trial-averaged calcium response for RSC axonal boutons, plotted in separate axes for each stimulation level, to show the variability in stimulation-evoked responses of single axonal boutons. These plots are the full distribution of data used to generate panel (d).

(f) Trial-averaged calcium response for dendritic spines in Cg1/M2, plotted in separate axes for each stimulation level, to show the variability in stimulation-evoked responses of single dendritic spines. These plots are the full distribution of data used to generate panel Fig. 4d.

(g) Individual mouse data for stimulation-induced calcium responses in spines in Cg1/M2. Five individual mice, with each mouse showing ΔF/F responses pre-saline, post-saline, pre-ketamine and post-ketamine.


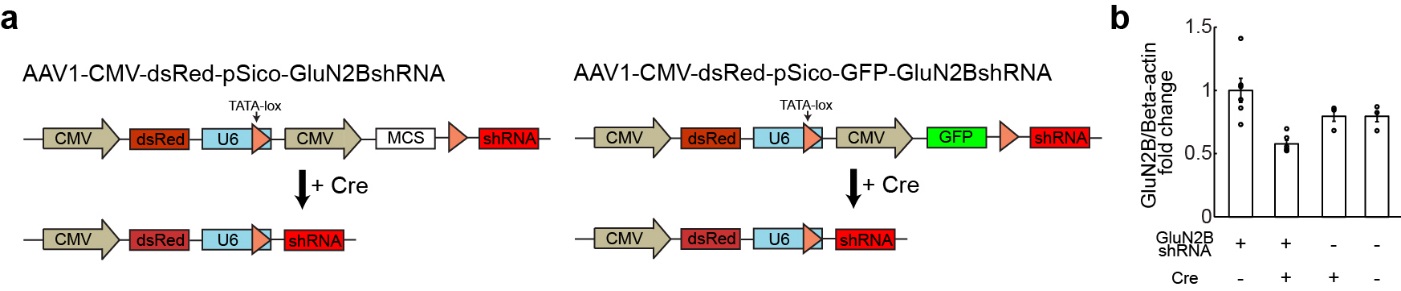


**Supplementary Fig. 6 Validation of Cre-dependent knockdown GluN2B receptors.**

(a) Left, schematic of AAV1-CMV-dsRed-pSico-GluN2BshRNA (standard AAV cassettes not drawn) for Cre-dependent expression of GluN2BshRNA used for combined knockdown and imaging experiments. Right, schematic of AAV1-CMV-dsRed-pSico-GFP-GluN2BshRNA for Cre-dependent expression of GluN2BshRNA used for behavioral experiments.

(b) Protein expression levels via Western blot to validate Cre-dependent GluN2B knockdown for AAV1-CMV-dsRed-pSico-GluN2BshRNA. GluN2B signals relative to beta-actin signals for 4 conditions (all values normalized to the GluN2BshRNA, no Cre condition): GluN2BshRNA, no Cre (1.00 ± 0.09, mean ± s.e.m.); GluN2BshRNA, Cre (0.58 ± 0.03), no GluN2BshRNA, Cre (0.80 ± 0.06); no GluN2BshRNA, no Cre (0.79 ± 0.06).
