# Supplementary material for: Ketamine disinhibits dendrites and enhances calcium signals in prefrontal dendritic spines
Source: Nat Commun. 2020 Jan 7;11:72. doi: 10.1038/s41467-019-13809-8 (PMC6946708; doi:10.1038/s41467-019-13809-8)
Supplement: Supplementary file 3 — Reporting Summary [file 41467_2019_13809_MOESM3_ESM.pdf]

nature research

Corresponding author(s): Alex C. Kwan
Last updated by author(s): Nov 7, 2019

# Reporting Summary

Nature Research wishes to improve the reproducibility of the work that we publish. This form provides structure for consistency and transparency in reporting. For further information on Nature Research policies, see Authors & Referees and the Editorial Policy Checklist.

## Statistics

For all statistical analyses, confirm that the following items are present in the figure legend, table legend, main text, or Methods section.

| n/a | Confirmed | |
|---|---|---|
| ☐ | ☒ | The exact sample size ($n$) for each experimental group/condition, given as a discrete number and unit of measurement |
| ☐ | ☒ | A statement on whether measurements were taken from distinct samples or whether the same sample was measured repeatedly |
| ☐ | ☒ | The statistical test(s) used AND whether they are one- or two-sided<br>*Only common tests should be described solely by name; describe more complex techniques in the Methods section.* |
| ☒ | ☐ | A description of all covariates tested |
| ☐ | ☒ | A description of any assumptions or corrections, such as tests of normality and adjustment for multiple comparisons |
| ☐ | ☒ | A full description of the statistical parameters including central tendency (e.g. means) or other basic estimates (e.g. regression coefficient) AND variation (e.g. standard deviation) or associated estimates of uncertainty (e.g. confidence intervals) |
| ☐ | ☒ | For null hypothesis testing, the test statistic (e.g. $F$, $t$, $r$) with confidence intervals, effect sizes, degrees of freedom and $P$ value noted<br>*Give P values as exact values whenever suitable.* |
| ☒ | ☐ | For Bayesian analysis, information on the choice of priors and Markov chain Monte Carlo settings |
| ☒ | ☐ | For hierarchical and complex designs, identification of the appropriate level for tests and full reporting of outcomes |
| ☒ | ☐ | Estimates of effect sizes (e.g. Cohen's $d$, Pearson's $r$), indicating how they were calculated |

*Our web collection on statistics for biologists contains articles on many of the points above.*

## Software and code

Policy information about availability of computer code

| | |
|---|---|
| Data collection | ScanImage v3.8 - for two photon imaging<br>Presentation v16 - control software for imaging and behavioral experiments<br>RHD2000 Interface - electrophysiology<br>VideoFreeze 2.6 - fear conditioning and locomotion<br>SR Lab - PPI |
| Data analysis | ImageJ v1.48 - general image processing<br>Turbo Reg plugin in ImageJ: motion correction<br>Matlab 2013b or 2017b - all analyses (see below)<br>NormCorre library in Matlab - motion correction<br>Custom scripts in Matlab - all imaging and behavior statistical analysis |

For manuscripts utilizing custom algorithms or software that are central to the research but not yet described in published literature, software must be made available to editors/reviewers. We strongly encourage code deposition in a community repository (e.g. GitHub). See the Nature Research guidelines for submitting code & software for further information.

## Data

Policy information about availability of data

All manuscripts must include a data availability statement. This statement should provide the following information, where applicable:

- Accession codes, unique identifiers, or web links for publicly available datasets
- A list of figures that have associated raw data
- A description of any restrictions on data availability

The data that support the findings of this study are available from the corresponding author upon reasonable request.

# Field-specific reporting

Please select the one below that is the best fit for your research. If you are not sure, read the appropriate sections before making your selection.

☒ Life sciences ☐ Behavioural & social sciences ☐ Ecological, evolutionary & environmental sciences

For a reference copy of the document with all sections, see nature.com/documents/nr-reporting-summary-flat.pdf

# Life sciences study design

All studies must disclose on these points even when the disclosure is negative.

| | |
|---|---|
| Sample size | No statistical tests were used to predetermine sample sizes, but sample sizes for this study are similar to those generally employed in the field. |
| Data exclusions | Animals were excluded from imaging experiments if the field of view did not permit collection of high quality data (e.g., poor optical clarity, excessive motion). |
| Replication | All experiments had at least 3 animals from at least 2 different litters ensure replicability across individuals and litters. |
| Randomization | Animals were assigned to ketamine or saline randomly. |
| Blinding | Experimenters were not blind to treatment and genotype during actual imaging, physiology and behavior experiments. However, all subsequent data processing were done either blind to treatments/genotype or performed automatically using scripts with consistent pre-established values. |

# Reporting for specific materials, systems and methods

We require information from authors about some types of materials, experimental systems and methods used in many studies. Here, indicate whether each material, system or method listed is relevant to your study. If you are not sure if a list item applies to your research, read the appropriate section before selecting a response.

## Materials & experimental systems

| n/a | Involved in the study | |
|---|---|---|
| ☐ | ☒ | Antibodies |
| ☒ | ☐ | Eukaryotic cell lines |
| ☒ | ☐ | Palaeontology |
| ☐ | ☒ | Animals and other organisms |
| ☒ | ☐ | Human research participants |
| ☒ | ☐ | Clinical data |

## Methods

| n/a | Involved in the study | |
|---|---|---|
| ☒ | ☐ | ChIP-seq |
| ☒ | ☐ | Flow cytometry |
| ☒ | ☐ | MRI-based neuroimaging |

## Antibodies

| | |
|---|---|
| Antibodies used | GluN2B: #4207S, Cell Signaling, 1:1000, rabbit polyclonal antibody<br>beta-actin: #4967S, Cell Signaling, 1:1000, rabbit polyclonal antibody |
| Validation | Validation by manufacturer and previous publications |

## Animals and other organisms

Policy information about studies involving animals; ARRIVE guidelines recommended for reporting animal research

| | |
|---|---|
| Laboratory animals | Only male mice (Mus musculus) were used.<br>For ketamine model<br>Adult (> 10 weeks of age) C57BL/6J (Jackson Laboratory Stock No. 000664)<br>Adult (> 10 weeks of age) SST-IRES-Cre (Jackson Laboratory Stock No. 013044) |
| Wild animals | No wild animals were used in study. |
| Field-collected samples | No field collected samples were used in study. |
| Ethics oversight | Institutional Animal Care and Use Committee, Yale University |

Note that full information on the approval of the study protocol must also be provided in the manuscript.
