# Supplementary material for: Ketamine disinhibits dendrites and enhances calcium signals in prefrontal dendritic spines
Source: Nat Commun. 2020 Jan 7;11:72. doi: 10.1038/s41467-019-13809-8 (PMC6946708; doi:10.1038/s41467-019-13809-8)
Supplement: Supplementary file 2 — Peer Review [file 41467_2019_13809_MOESM2_ESM.pdf]

Reviewers' comments:

Reviewer #1 (Remarks to the Author):

The manuscript by Ali et al. investigates the mechanisms by which systemic ketamine may mediate its effects through SST interneurons (INs) in the medial prefrontal cortex (mPFC). The authors attempt to fill a gap in current knowledge by focusing on SST INs and find that subanesthetic doses of ketamine suppress SST IN activity in the mPFC, which they argue results in disinhibition of postsynaptic pyramidal cell dendrites. They further perform targeted manipulation of NMDAR signaling in mPFC SST INs to show the impact on certain behaviors and inter-regional functional connectivity. The authors' work on elucidating some of the neurobiological mechanisms of action of sub-anesthetic ketamine is timely, important, and of broad interest, given the recent FDA approval of an antidepressant treatment based on ketamine, and the rapidly growing literature demonstrating ketamine's efficacy as a rapid-acting antidepressant that can be beneficial even for treatment-resistant patients. In their studies, the authors use and integrate powerful technologies such as 2-photon activity imaging of mPFC cells, boutons, and spines in behaving mice, targeted shRNA knockdown of SST IN NMDARs, and dual site LFP recordings. The authors' claims regarding the local mPFC circuit alterations induced by ketamine acting upon SST INs are novel and complement a recently published paper from the Liston laboratory focusing on ketamine's actions on PFC pyramidal cell ensembles and spine formation in chronic stress/depression animal models (Moda-Savak, Murdock, Parekh et al. 2019, Science – of note, though the latter paper was only published in the past month, it would be important for the authors to cite this work and comment on how it may fit with their studies and interpretations).

Together, the authors' main claims are quite convincing in many regards, there are some major concerns. First, the authors seem to motivate the work both on grounds of ketamine's initial psychotomimetic effects and its antidepressant effects. The former effects are well known and relatively better characterized, and the authors look at general behaviors (hyperlocomotion, PPI) which are (non-specifically) related to this. One area where the paper could have been significantly strengthened would have been to relate the authors' proposed mechanism of action more specifically to rescue of depression-related behaviors. Though timely and interesting the study is presently also flawed by many problems related to experimental design and analyses, making the interpretation of the results in many points ambiguous. These issues should be carefully (both experimentally and analytically) addressed before moving forward.

Major points:

1. Given the strong initial motion effects during the psychotomimetic period of ketamine in Fig. 1, is there any correlation between animal movement and subsequent mPFC cell type activity? Fig 1F: How consistent is this effect across mice? Does this magnitude of the activity rate change for each mouse correlate with its initial behavioral reaction (motion magnitude 5-30 min). If so, how would the authors interpret this?
2. Fig 1F: Since the author's choice of event detection algorithm can yield multiple events for a single time step (due to a larger amplitude), it is unclear whether the increase in activity rate is due to more frequent events or larger events (this appears to be broken down in the analysis for later experiments though?).
3. Fig 1H: Similar points on data presentation and analysis apply as raised above
4. For Fig 1 results generally, are the activity changes occurring mainly through locomotion-associated activity or during immobility, or just generally? One way the authors could analyze this is to calculate the activity rate change separately for different periods of movement intensity.
5. Relatedly in Fig 1, what are the temporal dynamics of these changes over the 30 min post-recording period? Is the effect long-lasting?
6. It would be helpful for the authors to comment on the validity of the 'peeling' algorithm event detection approach when applied to non-pyramidal cell body sources such as SST INs (Fig 1) and synaptic boutons (Fig 2).

7. Many of the cumulative probability histograms in Figs 1-3 do not appear normal? It would be more appropriate for the authors to conduct a non-parametric statistical test (e.g. K-S test)? The summary changes across mice could be reported as an average of means of individual mice, which is likely to follow a normal distribution.
8. In Fig S1: If the threshold is being calculated across ALL image frames, wouldn't this result in systematically different thresholds for saline vs ketamine conditions? This may select for spines showing only the largest events post-ketamine? It seems calculating the threshold based on the Pre-injection frames only should lead to comparable thresholds in both groups, for example.
9. Fig 2, similar points on data presentation and analysis apply as raised above, particularly confirming mouse-level replication since it is highly likely many of these axons originate from the same cells.
10. Fig 2, the data also appear to be highly skewed, with a long positive tail, which complicates the use of normal statistics and summarizing the data by average % changes in the text. For example, in 2D the authors report a 43.42% increase for the ketamine group vs 4.34% for saline, but the median % changes in the CDF plotted are in fact much closer together (within 5-10% of each other by eye), reflecting the bias of the long positive tail in estimating the mean of the distribution.
11. In general, for the % change calculations in Figs 1-3, won't normalizing by Pre-treat activity increases and decreases asymmetrically, as cells that did not fire until Post will have very high % increases from Pre (possibly infinite!), while a cell can only decrease its activity by 100%? This appears to greatly exaggerate the effect size (e.g., in 2e, half of the ROIs are decreasing or increasing their amplitude for both groups, yet a significant increase is reported for the ketamine group). Could the authors present the data as a simple change in activity rate, or report the raw activity rate data?
12. Fig 4D, what is Ca amplitude? dF/F?
13. Fig 4D, is the subject in the ANOVA mice or boutons? How consistent is the effect across mice?
14. In Fig. 5, the authors perform an SST specific NMDAR signaling knockdown and show this occludes ketamine's effects on SST INs. It would have been nice if the author's had also shown the occlusion effect with direct synaptic stimulation (as per Fig. 4). In addition to their Western Blot confirmation (Fig. S3), it would have also been nice if the authors would have also directly quantified how much their shRNA construct actually reduced NMDAR-mediated currents in SST INs. Fig 5, did the authors similarly measure mobility during these experiments? Was movement similar between groups? Does the effect replicate across individual mice?
15. In Fig. 5, does the shRNA knockdown cause a similar behavioral effect with initial hypermobility (as in Fig 1)?
16. In Fig. 6: Ketamine on its own blocks tFC; thus given the authors claim that ketamine is acting through PFC SST NMDARs, one would expect that reducing SST-NMDAR signaling with their shRNA approach should similarly result in disrupted tFC. Strangely however, this SST-NMDAR knockdown suddenly results in normal trace fear acquisition (in saline conditions) that is now insensitive to ketamine? The authors do not address this discrepancy with their proposed model of ketamine's actions.
17. In Fig. 6b, the authors plot freezing as a difference between CS+ and CS- presentations, it is unclear therefore whether the 'freezing deficit' is due to a problem in learning the CS+-US association (decreased freezing to the CS+ itself) or due to overgeneralization (increased freezing to CS-), for example. Is this impairment due to decreased freezing in CS+, or increased freezing in CS-?
18. As stated above, it would have been nice if the authors had also tested behaviors more specifically related to depression or memory deficits in psychotic disorders (e.g. T-maze, other spatial working memory tasks)
19. Fig. 7: Would have been nice if the authors had shown time dependence of their functional connectivity effects. Given that altered functional connectivity has been proposed in ketamine-induced psychosis and early schizophrenia, do they mirror the psychotomimetic behavioral phenotype time course of ketamine? If they persist longer than this how would the authors explain the relation to psychotomimetic effects?

Minor Comments:

1. Line 39: 'subjects' should state 'patients suffering from major depressive episodes'
2. Scale bars for zoomed insets in Fig 1e, 1h?
3. Line 104: 'while suppresses' should be 'while suppressing'

Reviewer #2 (Remarks to the Author):

Ali et al., present important new findings that relate the action of systemic ketamine treatment on dendritic disinhibition in the PFC and PFC dependent behaviors.

The comments below are selectively related to the use of transgenic mice and the behavioral experiments.

Based on the Jax stock number provided the transgenic mice referred to as "SST-Cre mice" would be more appropriately referred to as "SST-IRES-Cre mice". The genetic background of the SST-Cre-mice used is not described in the methods section. Based on the Jax stock number provided, the strain used results from the interbreeding of mice on a mixed C57/129 background. The same Sst-IRES-Cre knock-in allele is also currently available as C57BL/6N-congenic (Stock No. 018973) and C57BL/6J-congenic (Stock No. 028864). Therefore, in addition to providing the Jax stock number, the authors should state the genetic background of the mice used. This will help prevent any potential confusion over the background of the mice used. It is also important to state that the background of the mice is Not standard C57BL/6J, as might be assumed because wild type C57BL/6J mice are also used in this study.

The behavioral methods are clear and include adequate detail to replicate the studies. The behavioral experiments are well executed and the presentation of the data and statistical methods applied are appropriate.

The effects of ketamine with or without bilateral GluN2B-SST KD on trace fear conditioning, pre-pulse inhibition, and locomotor activity are reported.

In the methods section the ketamine dose described for fear conditioning and locomotor activity experiments are reported as 10mg/kg but the dose for pre-pulse inhibition is reported to be 40mg/kg. Is this a typographical error?

The finding that trace, but not delay conditioning is disrupted by ketamine is novel, as is the rescue of the effect of ketamine on trace conditioning by bilateral GluN2B-SST KD.
Rescue of the effect of ketamine on PPI by bilateral GluN2B-SST KD is also novel.

These experiments validate that the mechanism of GluN2B-SST KD to negate dendritic inhibition due to ketamine treatment observed using physiological/imaging methods is relevant to the behavioral effect of ketamine. Given the current application of subanesthetic dose of ketamine in clinical settings, this study and others that investigate the mechanisms of action are of significant interest.

Eleanor H. Simpson

Reviewer #3 (Remarks to the Author):

The manuscript by Ali et al. uses 2-photon imaging to show that acute ketamine administration increases the activity of prefrontal pyramidal neurons by reducing Sst-mediated inhibition of dendritic spines. Overall, the basic logic is sound and the data are convincing. Using GluN2B KD in Sst

interneurons to reproduce and occlude the effects of ketamine is particularly informative.

I found this manuscript very interesting to read, and think that it may provide a mechanism underlying some recently published findings about the antidepressant effects of ketamine (Moda-Sava et al., Sceicne, 2019). Therefore I would very much like to see this work published. That being said, there are two main issues I have with the manuscript in its current form, which should be addressed.

1. The behavioral data in Figure 6 is confusing. In Fig. 6b (Fear learning) and Fig. 6c (PPI), GluN2B KD in Sst interneurons blocks, but does not reproduce, the effects of ketamine. This doesn't make sense. The KD reproduces and occludes the effects of ketamine on Ca2+ events in pyramidal neuron dendrites and Sst interneurons, so the same should be true here. One could argue that there is some kind of subacute compensation which restores fear learning and PPI, even though the loss of GluN2B receptors in Sst interneurons eliminates the acute effects of ketamine -- however, this argument is a very hand-waving type of argument, and the current result is much less compelling than showing actual occlusion. Thus, as it stands, these results are almost uninterpretable.

2. The authors are using a dose of ketamine (10 mg/kg) which many labs have found elicits antidepressant effects. The work is motivated in large part by the antidepressant effects of ketamine, and a great deal of interest will be in how this relates to the antidepressant mechanism of action of ketamine. In this context, the authors have not examined whether this dose, in their hands, elicits effects in commonly used assays of antidepressant action (e.g., the forced swim test and tail suspension test), and/or whether these effects are occluded by GluN2B knockdown. I can imagine the authors might argue that they are more interested in the psychotomimetic effects of ketamine, or that these assays for antidepressant action are problematic. Both of these statements would be true, yet understanding how ketamine elicits effects in these assays is a question of central importance in the field, and addressing this question is what will make this paper so interesting to many readers. Perhaps the authors wanted to get this paper out quickly in light of the Moda-Sava publication, but looking at one antidepressant assay (either TST ot FST) doesn't seem like it would take too long.

Given that the current behavioral results are not compelling, and that it would be of great interest to explore how the mechanism being proposed here relates to the antidepressant actions of ketamine, I would recommend the authors to include some data on antidepressant assays in this study. Otherwise, the only other way I can think of, to make the current behavioral results on fear learning and PPI more compelling, would be to repeat these assays at shorter intervals after the injection of virus to KD GluN2B. This may reveal timepoints at which the KD does reproduce the behavioral effects of ketamine. Without more strongly tying the mechanism being studied here to a behavioral effect, this paper may end up being more appropriate for a more specialized journal.

Reviewer #1:

The manuscript by Ali et al. investigates the mechanisms by which systemic ketamine may mediate its effects through SST interneurons (INs) in the medial prefrontal cortex (mPFC). The authors attempt to fill a gap in current knowledge by focusing on SST INs and find that subanesthetic doses of ketamine suppress SST IN activity in the mPFC, which they argue results in disinhibition of postsynaptic pyramidal cell dendrites. They further perform targeted manipulation of NMDAR signaling in mPFC SST INs to show the impact on certain behaviors and inter-regional functional connectivity. The authors' work on elucidating some of the neurobiological mechanisms of action of sub-anesthetic ketamine is timely, important, and of broad interest, given the recent FDA approval of an antidepressant treatment based on ketamine, and the rapidly growing literature demonstrating ketamine's efficacy as a rapid-acting antidepressant that can be beneficial even for treatment-resistant patients.
In their studies, the authors use and integrate powerful technologies such as 2-photon activity imaging of mPFC cells, boutons, and spines in behaving mice, targeted shRNA knockdown of SST IN NMDARs, and dual site LFP recordings. The authors' claims regarding the local mPFC circuit alterations induced by ketamine acting upon SST INs are novel and complement a recently published paper from the Liston laboratory focusing on ketamine's actions on PFC pyramidal cell ensembles and spine formation in chronic stress/depression animal models (Moda-Savak, Murdock, Parekh et al. 2019, Science – of note, though the latter paper was only published in the past month, it would be important for the authors to cite this work and comment on how it may fit with their studies and interpretations).

RESPONSE: We appreciate the reviewer's positive view of our manuscript. The paper by Moda-Sava, Murdock, Parekh, et al., was published while this manuscript was under submission. In particular, we note that they have provided *in vivo* evidence for ketamine-induced increase in dendritic spine density. Moreover, Moda-Sava and colleagues discovered that ketamine restores coordinated activity in prefrontal ensembles, which is very interesting because SST interneurons, which our study implicates, are known to connect densely with pyramidal networks (Fino & Yuste. *Neuron* (2011)) and thereby regulate their correlated firing (Berger et al., *PLoS Biology* (2010)). We have now added the citations and discussed these points (lines 313 – 315, 325 – 327).

Together, the authors' main claims are quite convincing in many regards, there are some major concerns. First, the authors seem to motivate the work both on grounds of ketamine's initial psychotomimetic effects and its antidepressant effects. The former effects are well known and relatively better characterized, and the authors look at general behaviors (hyperlocomotion, PPI) which are (non-specifically) related to this. One area where the paper could have been significantly strengthened would have been to relate the authors' proposed mechanism of action more specifically to rescue of depression-related behaviors. Though timely and interesting the study is presently also flawed by many problems related to experimental design and analyses, making the interpretation of the results in many points ambiguous. These issues should be carefully (both experimentally and analytically) addressed before moving forward.

Major points:

1. Given the strong initial motion effects during the psychotomimetic period of ketamine in Fig. 1, is there any correlation between animal movement and subsequent mPFC cell type activity? Fig 1F: How consistent is this effect across mice? Does this magnitude of the activity rate change for each mouse correlate with its initial behavioral reaction (motion magnitude 5-30 min). If so, how would the authors interpret this?

RESPONSE: The initial hyperlocomotion effect due to systemic administration of ketamine was typical across mice (Fig. 1c, 6d). Fortunately, the effect was transient, and therefore we imaged from 30 – 60 minutes post-injection to avoid the potential influence of elevated movements on cortical neural activity. To more directly address the reviewer's point, we have gone back to the data for a re-analysis to ask if there was any correlation between the animal's motion (as measured during 5 – 30 min) and the mean or median ketamine-induced change in pyramidal cell activity (as imaged during 30 – 60 min) on an animal-by-animal basis. The correlation coefficients were not significantly different from zero ($P > 0.05$).

2. Fig 1F: Since the author's choice of event detection algorithm can yield multiple events for a single time step (due to a larger amplitude), it is unclear whether the increase in activity rate is due to more frequent events or larger events (this appears to be broken down in the analysis for later experiments though?). 3. Fig 1H: Similar points on data presentation and analysis apply as raised above

RESPONSE: The reviewer is correct that the event detection algorithm can produce multiple events for a single time step. For this reason, we did a further analysis to ask if ketamine influences amplitude (mean number of calcium events per frame, for frames with at least one event) and/or frequency (number of frames with at least one event divided by the duration of the imaging session) of the calcium signals. This analysis was done for dendritic calcium signals in the initial manuscript (Fig. 2e), and we have now applied it also to somatic calcium signals from pyramidal and SST neurons for the revision. For pyramidal neurons, ketamine increased the frequency and amplitude of calcium events. For SST interneurons, ketamine significantly decreased the frequency of calcium events relative to saline. The changes in frequency of calcium events for the two cell types are significant ($P = 3 \times 10^{-8}$ and 1×10^{-6}) and consistent with the interpretation that ketamine elevates pyramidal activity and reduces SST interneuron activity. The change in amplitude of calcium event for pyramidal neuron is more marginal ($P = 0.03$) and may reflect more spikes occurring together in the time scale of ~300 ms, limited by our imaging frame rate. The results are included as Supplementary Fig. 1a, c and cited in the main text (lines 95 and 105).

4. For Fig 1 results generally, are the activity changes occurring mainly through locomotion-associated activity or during immobility, or just generally? One way the authors could analyze this is to calculate the activity rate change separately for different periods of movement intensity.

RESPONSE: For a subset of data, we have recorded animal motion during the entire imaging session. This allowed us to analyze ketamine effects on synaptic calcium signals during stationary and moving periods. Pyramidal neuron activity was elevated and SST interneuron activity was reduced following ketamine during stationary periods, therefore indicating

movements are not the factor contributing to the observed neural activity changes. We have included the results in Supplementary Fig. 1b, d and cited in the main text (lines 96 – 97, 105).

5. Relatedly in Fig 1, what are the temporal dynamics of these changes over the 30 min post-recording period? Is the effect long-lasting?

RESPONSE: To address this question, we divided the 30 – 60 min post-injection imaging session into three 10-minute epochs, and determined ketamine's effect for each epoch. This analysis suggests that ketamine's effect on neural activity is not long-lasting in our hands. For both pyramidal and SST interneuron activity, the effect of ketamine relative to saline was statistically significant for 30 – 40 and 40 – 50 min epochs, but diminished over time and could no longer be detected for the 50 – 60 min epoch. We did the same analysis for SST axons and dendritic spines with similar results (Reviewer Fig. 1).

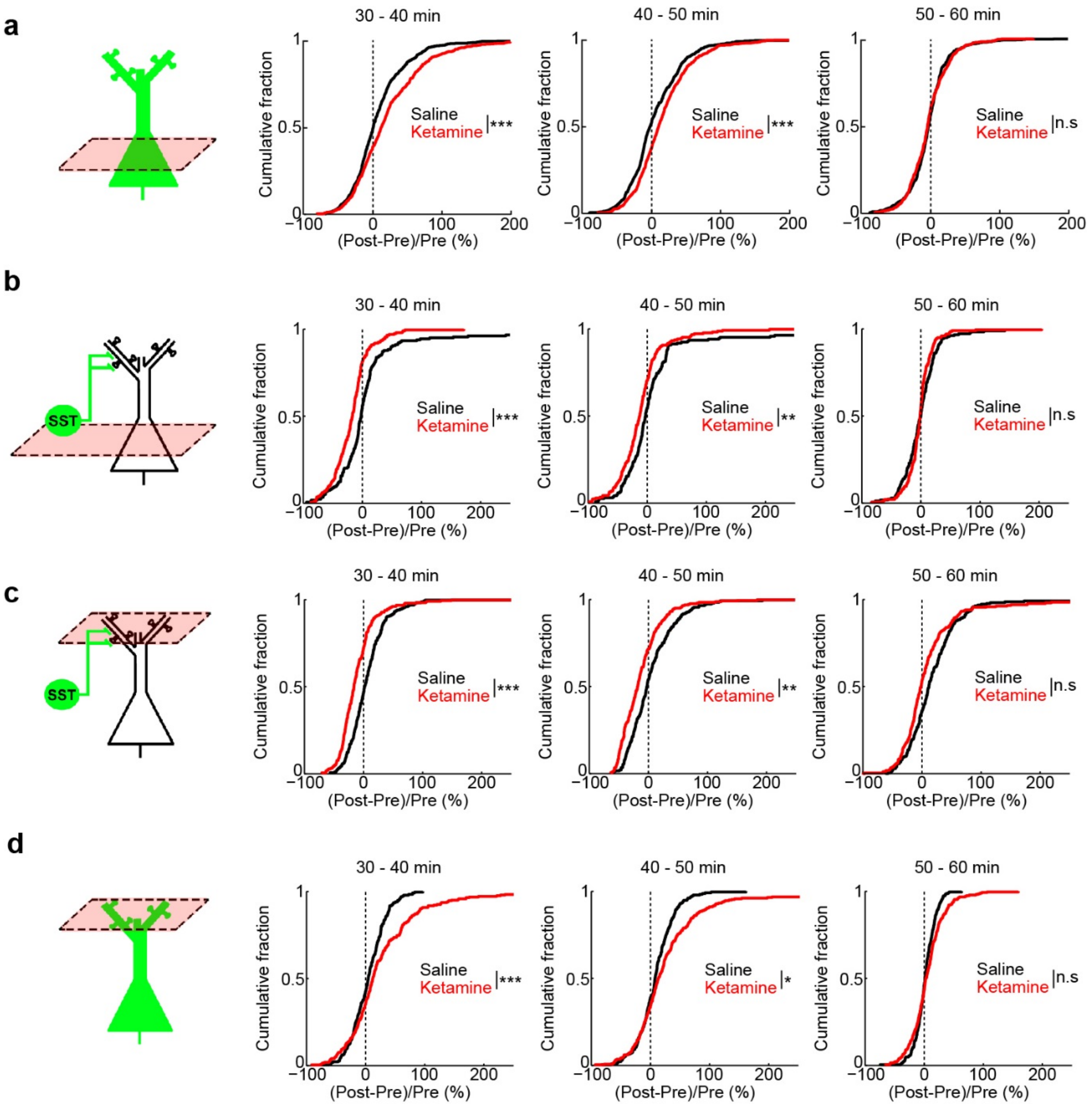

Reviewer Fig. 1 Time course of effects of ketamine on mPFC activity.
(a) Left, schematic of imaging of pyramidal neuron in Cg1/M2. Right, the normalized difference in the rate of spontaneous calcium events of pyramidal neurons at 30 – 40 min, 40 – 50 min and 50 – 60 min post-injection respectively.
(b) Same as (a) for SST interneuron.
(c) Same as (a) for SST axons.
(d) Same as (a) for dendritic spines.
** $P < 0.05$; ** $P < 0.01$; *** $P < 0.001$; n.s., not significant, two-sample t-test*

6. It would be helpful for the authors to comment on the validity of the 'peeling' algorithm event detection approach when applied to non-pyramidal cell body sources such as SST INs (Fig 1) and synaptic boutons (Fig 2).

RESPONSE: We decided to use the peeling algorithm on the basis of Lütcke et al. *Frontiers in Neural Circuits* (2013), which exhaustively tested the algorithm under a large range of imaging and neural conditions using simulated data. They found that the accuracy of the algorithm is dependent on several factors, most notably signal-to-noise and imaging frame rate. For our signal-to-noise ratio (≥3 based on ratio of peaks of calcium transients to SD of the whole distribution ΔF/F for the various cell-types/compartments) and imaging frame rate, the expected accuracy is a true positive rate of >80%. SST interneurons probably have a spike-related calcium response with different amplitude and decay time constant than pyramidal neurons. In this regard, Lütcke et al. has shown that detection can still be very reliable even if the amplitude and decay time constant of the template being used for the algorithm deviate by ~5% or ~1 s respectively from the true calcium response. Therefore, we believe the algorithm is appropriate for analyzing calcium signals from pyramidal and SST cell bodies.

For subcellular compartments such as dendritic spines and axonal boutons, we have applied the same peeling algorithm for consistency. We acknowledge, however, here that the peeling algorithm may be more prone to errors. For example, a prior study measured responses to a single action potential across multiple axonal boutons from the same neuron and found ten-fold variations in the intensity of calcium transients (Koester & Sakmann. *Journal of Physiology* (2000)). Moreover, each dendritic spine may have different unitary calcium responses to depolarization with distinct time constants (Kerlin et al., *Biorxiv* (2018)). Nevertheless, we believe these issues should not affect the conclusions for two reasons. One, we always make paired comparisons between the peeled output for ketamine versus the peeled output for saline, so all the data would go through the same algorithm. Two, we have analyzed signals from the dendritic spines using an alternative method. This method relies on threshold crossing, does not depend on templates, and yields similar conclusions. The analysis is included as Supplementary Fig. 3 and cited in the main text (lines 127 – 129).

7. Many of the cumulative probability histograms in Figs 1-3 do not appear normal? It would be more appropriate for the authors to conduct a non-parametric statistical test (e.g. K-S test)? The summary changes across mice could be reported as an average of means of individual mice, which is likely to follow a normal distribution.

RESPONSE: We re-ran all the statistical tests in Figs. 1-3 using the two-sample Kolmogorov–Smirnov (K-S) test. This nonparametric test allows us to compare the two cumulative fraction plots (saline versus ketamine) directly without any assumption of normality. The null hypothesis is that the two distributions come from the same underlying distribution. Below are the results with the *P*-values from the manuscript using t-tests shown in parentheses.

Fig. 1f (pyramidal neuron soma in mPFC): 2×10^{-8} (3×10^{-8})
Fig. 1i (SST soma in mPFC): 2×10^{-6} (1×10^{-4})
Fig. 2b (SST axonal boutons in mPFC): 2×10^{-9} (0.002)
Fig. 2d (dendritic spines in mPFC): 3×10^{-5} (0.02)
Fig. 3b (dendritic spines in M1): 0.004 (0.05)

As can be seen, none of the conclusions of whether to reject the null hypothesis at $\alpha = 0.05$ are affected by a change in statistical tests. We now report in the Methods section our finding of robustness to the type of statistical tests used (lines 725 – 730).

Regarding individual mice, to show the robustness at the subject-by-subject level, we now present the cumulative fraction plots for each and every mouse in Fig. 1-3. We used a total of 23 mice for the 5 types of cells/compartments imaged (mPFC: pyramidal neuron soma, SST interneuron soma, SST axonal boutons, dendritic spines; M1: dendritic spines). 20/23 mice showed ketamine effects (relative to saline) that were in the same direction as the overall effect in the main manuscript where all data were pooled. In other words, the vast majority of our overall results were consistent at the individual mouse level. The distributions for individual mice are now shown in a new Supplementary Fig. 2 and cited in the main text (lines 97, 105, 115, 123, 131).

In summary, our main results in Fig. 1-3 are robust to statistical tests and the effects are highly consistent at the individual mouse level.

8. In Fig S1: If the threshold is being calculated across ALL image frames, wouldn't this result in systematically different thresholds for saline vs ketamine conditions? This may select for spines showing only the largest events post-ketamine? It seems calculating the threshold based on the Pre-injection frames only should lead to comparable thresholds in both groups, for example.

RESPONSE: For each spine ROI, we used a total of 4 thresholds: 2 (drug: saline versus ketamine) x 2 (pre versus post). As suggested by the reviewer, we re-ran the analysis by computing the pre-saline threshold and then applying the same threshold to the post-saline data. We did the same for the ketamine condition. The statistical conclusions remain similar (Reviewer Fig. 2).

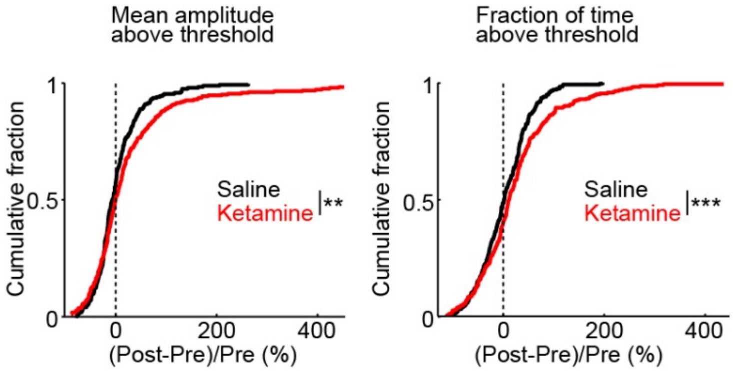

Reviewer Fig 2 Analyzing the calcium dynamics in dendritic spines using a pre-injection threshold and applying the same threshold to the post-injection data
** P < 0.05; ** P < 0.01; *** P < 0.001; n.s., not significant, two-sample t-test*

9. Fig 2, similar points on data presentation and analysis apply as raised above, particularly confirming mouse-level replication since it is highly likely many of these axons originate from the same cells.

RESPONSE: The reviewer is right that many of the boutons likely originate from the same axons/cells. For this reason, we modified a previously published procedure (Petreanu et al., *Nature*, (2012)) to prevent replicates from the same axons/cells from affecting the results. Briefly, the procedure involved using correlations in activity patterns to cluster the identified boutons and selecting a single randomly-selected bouton as representative of the cluster for use in subsequent analysis. This procedure is highly conservative and removed 50-80% of our originally identified boutons. Consequently, we believe that our axonal bouton results in Fig. 2a do not include replicates from the same axons/cells. This procedure is explained in the Methods section of the text (lines 536 – 556).

10. Fig 2, the data also appear to be highly skewed, with a long positive tail, which complicates the use of normal statistics and summarizing the data by average % changes in the text. For example, in 2D the authors report a 43.42% increase for the ketamine group vs 4.34% for saline, but the median % changes in the CDF plotted are in fact much closer together (within 5-10% of each other by eye), reflecting the bias of the long positive tail in estimating the mean of the distribution.

RESPONSE: The reviewer made an astute observation regarding the long positive tails of the distribution post-ketamine, which we similarly observed. We think this effect is physiological as it is consistent across individuals and different analyses (Supplementary Fig. 2; Fig. 2d-e). The interpretation is as follows: based on a number of anatomical studies, only 20-50% of cortical spines are innervated by SST axonal boutons (Chiu et al. *Science*, (2013); Villa et al. *Neuron*, (2016); Kwon et al. *Cerebral Cortex*, (2018)). If the effects of ketamine on spines are indeed mediated indirectly by inhibition of SST neurons, then we would expect only a subset of spines, and not all of them, to be disinhibited. This is what we see: ~30-50% of the distribution of spine activity has shifted post-ketamine, especially in the positive tails. Interestingly, for SST interneuron soma (Fig. 1i) and SST axonal boutons (Fig. 2b), the post-ketamine results instead showed a subtractive shift of the whole distribution. This is consistent with most, if not all, SST neurons having strong NMDAR currents (Wang et al. *Neuropsychopharmacology* (2009)). These

are important details to discuss, in addition to the initial report based on comparisons of the mean of the distributions. We now discuss these points in the text (lines 285 – 291).

11. In general, for the % change calculations in Figs 1-3, won't normalizing by Pre-treat activity increases and decreases asymmetrically, as cells that did not fire until Post will have very high % increases from Pre (possibly infinite!), while a cell can only decrease its activity by 100%? This appears to greatly exaggerate the effect size (e.g., in 2e, half of the ROIs are decreasing or increasing their amplitude for both groups, yet a significant increase is reported for the ketamine group). Could the authors present the data as a simple change in activity rate, or report the raw activity rate data?

RESPONSE: For analysis, we have normalized the activity change to each ROI's baseline activity, because baseline activity can differ by as much as an order of magnitude among ROIs. The (post-pre)/pre normalization takes this into account as to not overemphasize the cells with large baseline activity.

Following the reviewer's comment, we tested performing analyses based on (post-pre) difference in activity with no normalization. For example, for the main result in comparing synaptic calcium signals for ketamine versus saline injection, this alternate analysis yields many dendritic spines with large increases in event rates (Reviewer Fig. 3). The distribution is more skewed for no-normalization relative to normalization (compare with Fig. 2d). Specifically, this can be quantified by using a standard skewness measure (third standardized moment, $s = \frac{E(x-\mu)^3}{\sigma^3}$) to measure the degree of asymmetry in the distribution. For dendritic spines, this skewness measure was larger for the alternate analysis of simple difference (6.8) compared to our current analysis of normalized difference (2.9). Thus, we decided to not pursue the alternate analysis because it further skews the results to emphasize outliers and thus potentially influences statistical comparisons.

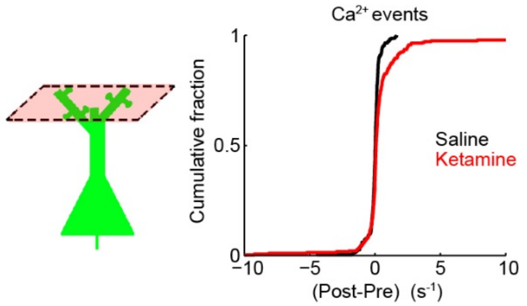

Reviewer Fig. 3 Change in activity of dendritic spines without normalization

12. Fig 4D, what is Ca amplitude? dF/F?

RESPONSE: Yes, it refers to dF/F. To avoid confusion, we have replaced the text labels with dF/F for Fig. 4d and Supplementary Fig. 4 accordingly.

13. Fig 4D, is the subject in the ANOVA mice or boutons? How consistent is the effect across mice?

RESPONSE: Fig. 4d refers to dendritic spines (not boutons). Dendritic spines were used as sample size in the statistical tests. The effect is consistent across mice: 4/5 mice displayed similar effects post-ketamine of increased stimulation-induced responses, especially for the higher stimulation frequencies. The plots for individual mice in this experiment have now been added to Supplementary Fig. 4g and cited in the main text (line 152).

14. In Fig. 5, the authors perform an SST specific NMDAR signaling knockdown and show this occludes ketamine's effects on SST INs. It would have been nice if the authors had also shown the occlusion effect with direct synaptic stimulation (as per Fig. 4). In addition to their Western Blot confirmation (Fig. S3), it would have also been nice if the authors would have also directly quantified how much their shRNA construct actually reduced NMDAR-mediated currents in SST INs.

RESPONSE: Our co-author Dr. Ronald Duman has a separate manuscript currently under review that uses the same AAV1-CMV-dsRed-pSico-GluN2BshRNA virus. They have performed slice electrophysiology to confirm the reduction of NMDAR-induced currents by ~50% in cortical SST interneurons expressing the GluN2BshRNA relative to control cells (Reviewer Fig. 4). Our data is consistent with this slice results, as we also showed functionally that GluN2BshRNA lowered the activity of SST interneurons *in vivo* (Fig. 5c). We have added a citation to the manuscript describing the slice results (lines 168 – 169).

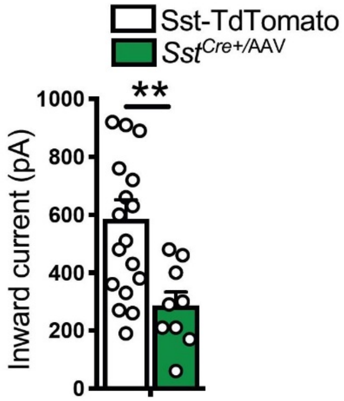

Reviewer Fig.4 Reduction of NMDAR-mediated currents in SST-Cre cells expressing GluN2BshRNA
There was a significant reduction in NMDA-induced inward currents in SST interneurons expressing Cre and GluN2BshRNA compared with control SST-TdTomato interneurons (two-sample t-test, P = 0.006). Each open circle is a cell.

Fig 5, did the authors similarly measure mobility during these experiments? Was movement similar between groups? Does the effect replicate across individual mice?
15. In Fig. 5, does the shRNA knockdown cause a similar behavioral effect with initial hypermobility (as in Fig 1)?

RESPONSE: GluN2B KD in prefrontal SST interneurons did not cause any hyperlocomotion at baseline, compared to non-KD (Fig. 6d). Note that each line is an individual mouse to illustrate the consistency of the null effect.

16. In Fig. 6: Ketamine on its own blocks tFC; thus given the authors claim that ketamine is acting through PFC SST NMDARs, one would expect that reducing SST-NMDAR signaling with their shRNA approach should similarly result in disrupted tFC. Strangely however, this SST-NMDAR knockdown suddenly results in normal trace fear acquisition (in saline conditions) that is now insensitive to ketamine? The authors do not address this discrepancy with their proposed model of ketamine's actions.

RESPONSE: Thank you for pointing out this important discrepancy, which motivated new experiments. Namely, the discrepancy arises because the GluN2B KD reduced SST interneuron activity (Fig. 5c), increased dendritic spine activity (Fig. 5e), and rendered ketamine ineffective on trace fear conditioning (Fig. 6a), however the GluN2B KD alone had no detectable effect on trace fear conditioning and PPI. We suspected the reason may be compensatory mechanisms. More specifically, behavioral testing occurred more than 4 weeks after GluN2B KD, and it is possible that other brain regions become involved to restore trace fear conditioning and PPI (Fanselow, *Trends in Cognitive Sciences*, (2010).

To test this hypothesis, we performed experiments in which we reduced SST interneuron activity acutely. The idea is that there would be no time for compensatory mechanisms to act, and therefore a deficit in trace fear conditioning. We used Cre-dependent expression of hM4D(Gi), a designer receptor exclusively activated by designer drug (DREADD) and SST-Cre mice. After waiting 4 weeks for the hM4D(Gi) to express in SST interneurons, we then repeated the same trace fear conditioning and pre-pulse inhibition experiments along with ketamine or saline treatment. CNO (in DMSO) or vehicle (saline in DMSO) was injected (i.p., 5 mg/kg) 30 min before trace fear conditioning followed by ketamine or saline 15 min before commencement of conditioning. For PPI experiments, the sequence was 30 min for CNO or vehicle followed by ketamine or saline before immediate commencement of PPI. Consistent with the compensatory hypothesis, acute reduction of SST interneuron activity via hM4D(Gi) + CNO impaired trace fear learning and PPI in saline-treated animals while ketamine did not cause any additional effects. We performed the controls to show that the effect was not due to hM4D(Gi) or CNO injection.

In summary, acute downregulation of mPFC SST interneuron activity fully reproduces the trace fear learning and pre-pulse inhibition effects of ketamine, but not chronic downregulation which allows the behaviors to persist although it still blocks additional ketamine's effects. These data provide a reasonable explanation for the discrepancy, and provides additional insights into the cellular mechanism. We have now included these data in the main manuscript (Fig. 7) and modified the methods (lines 457 – 460; 670 – 672; 692 – 694) and results sections accordingly (lines 202 – 220).

17. In Fig. 6b, the authors plot freezing as a difference between CS+ and CS- presentations, it is unclear therefore whether the 'freezing deficit' is due to a problem in learning the CS+-US association (decreased freezing to the CS+ itself) or due to overgeneralization (increased freezing to CS-), for example. Is this impairment due to decreased freezing in CS+, or increased freezing in CS-?

RESPONSE: The ketamine-induced reduction in learning was due to a decrease in freezing to CS+ with little effect on the animal's response to CS- (Reviewer Fig. 5).

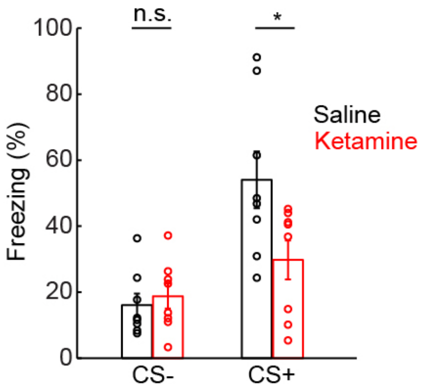

Reviewer Fig. 5 Effects of ketamine on freezing to CS- and CS+ during testing
** P < 0.05; ** P < 0.01; *** P < 0.001; n.s., not significant, Wilcoxon rank-sum test*

18. As stated above, it would have been nice if the authors had also tested behaviors more specifically related to depression or memory deficits in psychotic disorders (e.g. T-maze, other spatial working memory tasks)

RESPONSE: We agree that other behaviors such as those related to depression or memory would have been interesting. We are limited by the scope of the study. Currently, for behavior, we have included three assays (trace fear learning, pre-pulse inhibition, and locomotor activity) and two manipulations (shRNA-mediated knockdown and DREADD). The majority of the work is circuit-based dissection with optical imaging, and we believe that the behavioral work is a sufficient complement.

19. Fig. 7: Would have been nice if the authors had shown time dependence of their functional connectivity effects. Given that altered functional connectivity has been proposed in ketamine-induced psychosis and early schizophrenia, do they mirror the psychotomimetic behavioral phenotype time course of ketamine? If they persist longer than this how would the authors explain the relation to psychotomimetic effects?

RESPONSE: The local field potential (LFP) results were collected from 30 – 60 min following ketamine administration. To see if there is any time dependence, we re-analyzed the data in 10-minute epochs. The effect is more variable because each bin now contains less data, but nevertheless there may be some time dependence in the effect of ketamine on functional connectivity. Namely, the elevated connectivity was most prominent for the 30 – 40 min bin, but declines subsequently (Reviewer Fig. 6). The exact time course, and comparison with human studies, is more difficult as we have not accurately measured the pharmacokinetics to know the time course of intracortical ketamine concentration. Nevertheless, this current analysis suggests that the elevated functional connectivity is transient, consistent with psychosis being an acute effect of ketamine in humans.

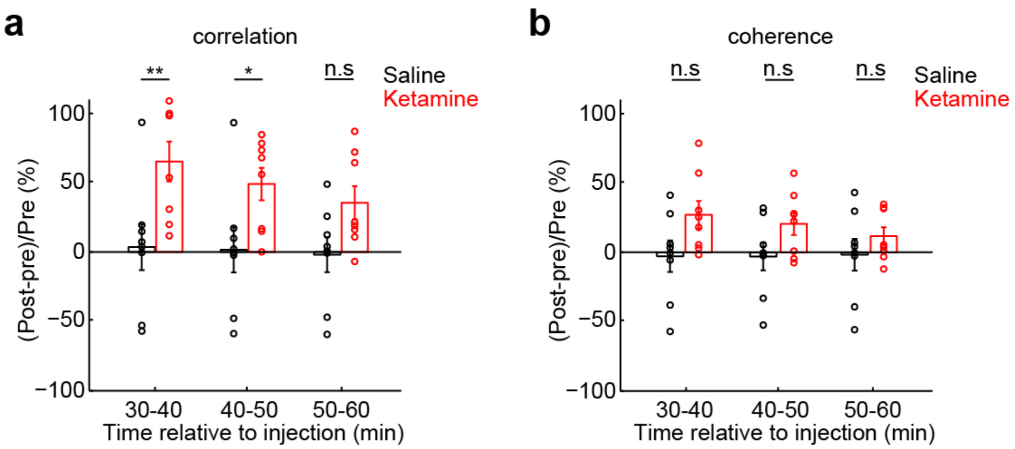

Reviewer Fig. 6 Time course of ketamine's effects on prefrontal cortical functional connectivity

(a) Functional connectivity after ketamine or saline for SST-Cre animals at 30-40 min, 40-50 min, and 50-60 min post-injection as measured by lag-zero correlation between integrated gamma band signals in Cg1/M2 and RSC.

(b) Same as (a) for coherence.

** $P < 0.05$; ** $P < 0.01$; *** $P < 0.001$; n.s., not significant, Wilcoxon rank-sum tests*

Minor Comments:

1. Line 39: 'subjects' should state 'patients suffering from major depressive episodes'

RESPONSE: Corrected.

2. Scale bars for zoomed insets in Fig 1e, 1h?

RESPONSE: Added. Added to Fig. 2a too.

3. Line 104: 'while suppresses' should be 'while suppressing'

RESPONSE: Corrected.

Reviewer #2:

Ali et al., present important new findings that relate the action of systemic ketamine treatment on dendritic disinhibition in the PFC and PFC dependent behaviors.

The comments below are selectively related to the use of transgenic mice and the behavioral experiments.

Based on the Jax stock number provided the transgenic mice referred to as "SST-Cre mice" would be more appropriately referred to as "SST-IRES-Cre mice".

RESPONSE: Thank you for the suggestion. We now refer to the animals as 'SST-IRES-Cre mice'.

The genetic background of the SST-Cre-mice used is not described in the methods section. Based on the Jax stock number provided, the strain used results from the interbreeding of mice on a mixed C57/129 background. The same Sst-IRES-Cre knock-in allele is also currently available as C57BL/6N-congenic (Stock No. 018973) and C57BL/6J-congenic (Stock No. 028864). Therefore, in addition to providing the Jax stock number, the authors should state the genetic background of the mice used. This will help prevent any potential confusion over the background of the mice used. It is also important to state that the background of the mice is Not standard C57BL/6J, as might be assumed because wild type C57BL/6J mice are also used in this study.

RESPONSE: We have added the following to the Method section "To selectively image or manipulate SST interneurons, we utilized adult male SST-Cre on a mixed C57BL/6 x 129S4/SvJae background" (lines 334 - 336). Note that the Jackson Laboratory website does not specify for this stock whether the background is a C57BL/6J or C57BL/6N sub-strain.

The behavioral methods are clear and include adequate detail to replicate the studies. The behavioral experiments are well executed and the presentation of the data and statistical methods applied are appropriate. The effects of ketamine with or without bilateral GluN2B-SST KD on trace fear conditioning, pre-pulse inhibition, and locomotor activity are reported. In the methods section the ketamine dose described for fear conditioning and locomotor activity experiments are reported as 10mg/kg but the dose for pre-pulse inhibition is reported to be 40mg/kg. Is this a typographical error?

RESPONSE: We used 40 mg/kg of ketamine for PPI, which was higher than the 10 mg/kg used for imaging and other behavioral experiments. This was because when we initially tested 10 mg/kg in control mice, ketamine did not yield reliable effects on PPI performance. We therefore followed other published papers that have used higher dosages in mice (e.g., 30 mg/kg (Chan et al., *Psychopharmacology*, (2008)). Having a robust effect of ketamine in control mice was important to avoid floor effects when moving on to test effect of ketamine in mice with SST GluN2B-KD.

The finding that trace, but not delay conditioning is disrupted by ketamine is novel, as is the rescue of the effect of ketamine on trace conditioning by bilateral GluN2B-SST KD.

Rescue of the effect of ketamine on PPI by bilateral GluN2B-SST KD is also novel. These experiments validate that the mechanism of GluN2B-SST KD to negate dendritic inhibition due to ketamine treatment observed using physiological/imaging methods is relevant to the behavioral effect of ketamine. Given the current application of subanesthetic dose of ketamine in clinical settings, this study and others that investigate the mechanisms of action are of significant interest.

RESPONSE: We thank the reviewer for recognizing the novelty and significance of the manuscript.

Reviewer #3:

The manuscript by Ali et al. uses 2-photon imaging to show that acute ketamine administration increases the activity of prefrontal pyramidal neurons by reducing Sst-mediated inhibition of dendritic spines. Overall, the basic logic is sound and the data are convincing. Using GluN2B KD in Sst interneurons to reproduce and occlude the effects of ketamine is particularly informative. I found this manuscript very interesting to read, and think that it may provide a mechanism underlying some recently published findings about the antidepressant effects of ketamine (Moda-Sava et al., Sceicne, 2019). Therefore I would very much like to see this work published. That being said, there are two main issues I have with the manuscript in its current form, which should be addressed.

RESPONSE: We are pleased that the reviewer finds the logic sound and data convincing. We thank the reviewer for bringing up the Moda-Sava, Murdock, Parekh, et al., Science, 2019 paper, which was published while our manuscript was under submission. In particular, we note that they have provided *in vivo* evidence for ketamine-induced increase in dendritic spine density. Moreover, Moda-Sava and colleagues discovered that ketamine restores coordinated activity in prefrontal ensembles, which is very interesting because SST interneurons, which our study implicates, are known to connect densely with pyramidal networks (Fino & Yuste. *Neuron* (2011)) and thereby regulate their correlated firing (Berger et al., *PLoS Biology* (2010)). We have now added the citations and discussed these points (lines 313 – 315, 325 – 327).

1. The behavioral data in Figure 6 is confusing. In Fig. 6b (Fear learning) and Fig. 6c (PPI), GluN2B KD in Sst interneurons blocks, but does not reproduce, the effects of ketamine. This doesn't make sense. The KD reproduces and occludes the effects of ketamine on Ca2+ events in pyramidal neuron dendrites and Sst interneurons, so the same should be true here. One could argue that there is some kind of subacute compensation which restores fear learning and PPI, even though the loss of GluN2B receptors in Sst interneurons eliminates the acute effects of ketamine -- however, this argument is a very hand-waving type of argument, and the current result is much less compelling than showing actual occlusion. Thus, as it stands, these results are almost uninterpretable.

RESPONSE: Thank you for pointing out this important discrepancy, which motivated new experiments. Namely, the discrepancy arises because the GluN2B KD reduced SST interneuron activity (Fig. 5c), increased dendritic spine activity (Fig. 5e), and rendered ketamine ineffective on trace fear conditioning and PPI (Fig. 6a), however the GluN2B KD alone had no detectable effect on trace fear conditioning. As the reviewer noted, a plausible reason is compensatory mechanisms. More specifically, behavioral testing occurred more than 4 weeks after GluN2B KD, and it is possible that other brain regions become involved to restore trace fear conditioning and PPI (Fanselow, *Trends in Cognitive Sciences*, (2010).

To test this hypothesis, we performed experiments in which we reduced SST interneuron activity acutely. The idea is that there would be no time for compensatory mechanisms to act, and therefore a deficit in trace fear conditioning. We used Cre-dependent expression of hM4D(Gi), a designer receptor exclusively activated by designer drug (DREADD) and SST-Cre mice. After waiting 4 weeks for the hM4D(Gi) to express in SST interneurons, we then repeated the same

trace fear conditioning and pre-pulse inhibition experiments along with ketamine or saline treatment. CNO (in DMSO) or vehicle (saline in DMSO) was injected (i.p., 5 mg/kg) 30 min before trace fear conditioning followed by ketamine or saline 15 min before commencement of conditioning. For PPI experiments, the sequence was 30 min for CNO or vehicle followed by ketamine or saline before immediate commencement of PPI. Consistent with the compensatory hypothesis, acute reduction of SST interneuron activity via hM4D(Gi) + CNO impaired trace fear learning and PPI in saline-treated animals while ketamine did not cause any additional effects. We performed the controls to show that the effect was not due to hM4D(Gi) or CNO injection.

In summary, acute downregulation of mPFC SST interneuron activity fully reproduces the trace fear learning and pre-pulse inhibition effects of ketamine, but not chronic downregulation which allows the behaviors to persist although it still blocks additional ketamine's effects. These data provide a reasonable explanation for the discrepancy, and provides additional insights into the cellular mechanism. We have now included these data in the main manuscript (Fig. 7) and modified the methods (lines 457 – 460; 670 – 672; 692 – 694) and results sections accordingly (lines 202 – 220).

2. The authors are using a dose of ketamine (10 mg/kg) which many labs have found elicits antidepressant effects. The work is motivated in large part by the antidepressant effects of ketamine, and a great deal of interest will be in how this relates to the antidepressant mechanism of action of ketamine. In this context, the authors have not examined whether this dose, in their hands, elicits effects in commonly used assays of antidepressant action (e.g., the forced swim test and tail suspension test), and/or whether these effects are occluded by GluN2B knockdown. I can imagine the authors might argue that they are more interested in the psychotomimetic effects of ketamine, or that these assays for antidepressant action are problematic. Both of these statements would be true, yet understanding how ketamine elicits effects in these assays is a question of central importance in the field, and addressing this question is what will make this paper so interesting to many readers.
Perhaps the authors wanted to get this paper out quickly in light of the Moda-Sava publication, but looking at one antidepressant assay (either TST ot FST) doesn't seem like it would take too long.

RESPONSE: Here our work mainly addresses the acute consequences of ketamine administration on the prefrontal cortical microcircuit. Three behavioral assays were selected based on the expected acute changes. To tackle the question on the longer-term antidepressant effects, the appropriate approach would require testing a large battery of anxiety and depression-related behaviors. This is because as the reviewer noted, any one assay for antidepressant action may be problematic. We believe the substantial behavioral work is beyond the scope of the study (which focuses on circuit dynamics using optical imaging).

That being said, we should note that the dendritic inhibition mechanism is likely to be involved in the antidepressant effects of ketamine. Our co-author, Dr. Ronald Duman, has a separate manuscript currently under peer review. In that manuscript, they described using the same shRNA viruses to test effects of GluN2B KD on pyramidal, SST, and PV interneurons for a battery of depression-related assays including forced swim test, novelty suppressed feeding test,

home cage feeding, and female urine sniffing test. One conclusion of that work is that prefrontal SST interneurons contribute to the antidepressive behavioral effects of ketamine. That work is distinct from our manuscript as it centers around depressive-like behaviors, with no *in vivo* imaging and did not characterize any neuronal or synaptic calcium signals. The manuscript is available upon request for the Editor to ensure there is no overlap.

Given that the current behavioral results are not compelling, and that it would be of great interest to explore how the mechanism being proposed here relates to the antidepressant actions of ketamine, I would recommend the authors to include some data on antidepressant assays in this study. Otherwise, the only other way I can think of, to make the current behavioral results on fear learning and PPI more compelling, would be to repeat these assays at shorter intervals after the injection of virus to KD GluN2B. This may reveal timepoints at which the KD does reproduce the behavioral effects of ketamine. Without more strongly tying the mechanism being studied here to a behavioral effect, this paper may end up being more appropriate for a more specialized journal.

In terms of relating the neural results to behavioral findings, the new DREADD experiments provided novel results to distinguish the acute versus subchronic effects of downregulating mPFC SST interneuron activity. The data provided empirical support towards the idea that the chronic nature of the downregulation was why there was a discrepancy in the initial manuscript. As a result, we believe that there is now a cohesive framework for interpreting the neural and behavioral findings in our study.

REVIEWERS' COMMENTS:

Reviewer #2 (Remarks to the Author):

Thank you for clarifying that the dose of Ketamine used for PPI experiments was 40mg/KG and for providing the rational for this higher dose, which is reasonable. However, given that the dose of ketamine used for all other experiments (behavioral and imaging) was 10mg/kg, this difference should be made more apparent. For example, in Figure 6, all data are labelled "saline" or "ketamine", but the dose is not the same for each figure in the panel. Doses are not reported in the results sections and the drugs subsection of the methods could be misleading as only a single working dose is provided. Please report the dose used in each experiment in the results section (text or figure). This is important information if one is considering the relationship between the results of the imaging and behavior experiments.

Reviewer #3 (Remarks to the Author):

I am satisfied with the author's response to my first major comment.

Re: my second major comment, that the authors should have included some data re: the antidepressant effects of the dose of ketamine used here and whether these are occluded by GluN2B knockdown. Reviewer 1 also made the same recommendation. The authors did not include that data. They argue that really, a full battery of behavioral assays would be required, and that this has been done in another manuscript by one of the authors. As I said in my original comment, a full battery of assays wouldn't be necessary, just one common antidepressant assay such as the FST or TST would suffice.
I gather that the authors do not want to include that information here, even though they have that data and it more or less shows what I asked about, because it is part of another manuscript. I can certainly understand and appreciate that. However, it does create a bit of a conundrum, because the main reason people would be interested in this paper is because of ketamine's antidepressant effects. The title of the paper is "Ketamine disinhibits dendrites and enhances calcium signals in prefrontal dendritic spines." If it were not for the antidepressant effects of ketamine, this would be a nice paper, but probably not of sufficiently high interest to be published in Nature Communications.
I will defer to the editors about how best to manage this issue. I really like this paper, but I also feel that information about the antidepressant relevance of the mechanisms being studied is important. Perhaps it would be sufficient to reference the result from the other paper.

REVIEWERS' COMMENTS:

Reviewer #2 (Remarks to the Author):

Thank you for clarifying that the dose of Ketamine used for PPI experiments was 40mg/KG and for providing the rational for this higher dose, which is reasonable. However, given that the dose of ketamine used for all other experiments (behavioral and imaging) was 10mg/kg, this difference should be made more apparent. For example, in Figure 6, all data are labelled "saline" or "ketamine", but the dose is not the same for each figure in the panel. Doses are not reported in the results sections and the drugs subsection of the methods could be misleading as only a single working dose is provided.

Please report the dose used in each experiment in the results section (text or figure). This is important information if one is considering the relationship between the results of the imaging and behavior experiments.

RESPONSE: We now report clearly the respective doses and rationale for using a higher dose for PPI in Methods (lines 446-449). The doses are also reported in Results for each experiment and in all figure legends that mention ketamine (Figures 1-8; Supplementary Figures 1-5).

Reviewer #3 (Remarks to the Author):

I am satisfied with the author's response to my first major comment.

Re: my second major comment, that the authors should have included some data re: the antidepressant effects of the dose of ketamine used here and whether these are occluded by GluN2B knockdown. Reviewer 1 also made the same recommendation. The authors did not include that data. They argue that really, a full battery of behavioral assays would be required, and that this has been done in another manuscript by one of the authors. As I said in my original comment, a full battery of assays wouldn't be necessary, just one common antidepressant assay such as the FST or TST would suffice.

I gather that the authors do not want to include that information here, even though they have that data and it more or less shows what I asked about, because it is part of another manuscript. I can certainly understand and appreciate that. However, it does create a bit of a conundrum, because the main reason people would be interested in this paper is because of ketamine's antidepressant effects. The title of the paper is "Ketamine disinhibits dendrites and enhances calcium signals in prefrontal dendritic spines." If it were not for the antidepressant effects of ketamine, this would be a nice paper, but probably not of sufficiently high interest to be published in Nature Communications.

I will defer to the editors about how best to manage this issue. I really like this paper, but I also feel that information about the antidepressant relevance of the mechanisms being studied is important. Perhaps it would be sufficient to reference the result from the other paper.

RESPONSE: We acknowledge the reviewer's comments. Our collaborator's manuscript has recently been accepted for publication, and will be cited accordingly in this paper (lines 169; 323).
